# 

**Published:** 1880-02

**Authors:** 


### CONTENTS FOR FEBRUARY, 1880.

ORIGINAL COMMUNICATIONS.

ART. I.—Flagellation of the Child's Back Previous to its Complete Delivery, as a Preventive of Uterine Hemorrhage, etc. By ISAAC E. TAYLOR, M. D. 53

ART. II.—Wire Gauze Supporter for the Treatment of Fractures of the Lower Extremities. BY HARVEY L. BYRD, M. D. .......... 71

ART. III.—On the Use of Hydrobromic Ether as an Aæsthetic for Surgical Operations. By LAWRENCE TURNBULL, M. D., Ph. G. .......... 74

ART. IV.—" Every Tooth Containing a Metallic Filling is a Galvanic Battery in Active Operation," etc. By HENRY S. CHASE, M. D., D. D. S. .......... 77

ART. V.—A Suspensory. By BASIL M. WILKERSON, M. D., D. D. S .......... 81

Translations .......... 82

ECLECTIC DEPARTMENT.

Musical Heart Murmurs. By J. M. DA COSTA, M. D. .......... 84

Miasm and Fevers .......... 87

Replantation of Teeth .......... 89

EDITORIAL DEPARTMENT.

Medical Hall for Baltimore .......... 91

Independent Journalism .......... 92

Associations, Fees, Contracts, etc. .......... 93

Translations .......... 95

The Practitioner .......... 95

The Underwriter .......... 96

Deaths from Chloroform .......... 96

BIBLIOGRAPHICAL DEPARTMENT .......... 96

EXCERPTA.

Transmission of Hydrophobia from Men to Rabbits .......... 100

The National Board of Health .......... 101

Medical Charity, and What Comes of It .......... 101

New Method of Preserving Dead Bodies .......... 102

Childbirth After Ovariotomy .......... 103

Diarrhœa a Zymotic Disease .......... 104

Treatment of Hepatic Calculi .......... 104

Value of Benzoate of Soda in Diphtheria .......... 105

German Therapeutics .......... 105

Physicians as Microscopists .......... 106

Restoration of the Menses .......... 106

Incontinence of Urine in Children .......... 107

Identification of the Prince Imperial .......... 108

Hymeneal, Natalitial, Necrological .......... 108

Cause of Delay of this Number .......... 110

Addendum and Errata .......... 110

Mortuary Statistics .......... 111

Meteorological Report for January .......... 113

A Letter of Individual Importance to Every one Interested in Dentistry .......... 113

